# Surface modification of TPGS-*b*-(PCL-*ran*-PGA) nanoparticles with polyethyleneimine as a co-delivery system of TRAIL and endostatin for cervical cancer gene therapy

**DOI:** 10.1186/1556-276X-8-161

**Published:** 2013-04-09

**Authors:** Yi Zheng, Hongbo Chen, Xiaowei Zeng, Zhigang Liu, Xiaojun Xiao, Yongqiang Zhu, Dayong Gu, Lin Mei

**Affiliations:** 1 The Shenzhen Key Lab of Gene and Antibody Therapy, Center for Biotechnology and BioMedicine and Division of Life Science and Health, Graduate School at Shenzhen, Tsinghua University, L401, Tsinghua Campus, Xili University Town, Shenzhen, Guangdong Province, 518055, People's Republic of China; 2 School of Life Sciences, Tsinghua University, Beijing, 100084, People's Republic of China; 3 School of Medicine, Shenzhen University, Shenzhen, 518060, People's Republic of China; 4 Institute of Disease Control and Prevention, Shenzhen Entry-Exit Inspection and Quarantine Bureau, Shenzhen, 518045, People's Republic of China

**Keywords:** TPGS-*b*-(PCL-*ran*-PGA), Nanoparticles, Cervical cancer, TRAIL, Endostatin, Gene delivery

## Abstract

The efficient delivery of therapeutic genes into cells of interest is a critical challenge to broad application of non-viral vector systems. In this research, a novel TPGS-*b*-(PCL-*ran*-PGA) nanoparticle modified with polyethyleneimine was applied to be a vector of tumor necrosis factor-related apoptosis-inducing ligand (TRAIL) and endostatin for cervical cancer gene therapy. Firstly, a novel biodegradable copolymer, TPGS-*b*-(PCL-*ran*-PGA), was synthesized and characterized. The nanoparticles were fabricated by an emulsion/solvent evaporation method and then further modified with polyethyleneimine (PEI) carrying TRAIL and/or endostatin genes. The uptake of pIRES2-EGFP and/or pDsRED nanoparticles by HeLa cells were observed by fluorescence microscopy and confocal laser scanning microscopy. The cell viability of TRAIL/endostatin-loaded nanoparticles in HeLa cells was assessed by 3-(4,5-dimethylthiazol-2-yl)-2,5-diphenyl-2*H*-tetrazolium bromide assay. Severe combined immunodeficient mice carrying HeLa tumor xenografts were treated in groups of six including phosphate-buffered saline control, blank TPGS-*b*-(PCL-*ran*-PGA) nanoparticles, blank TPGS-*b*-(PCL-*ran*-PGA)/PEI nanoparticles, and three types of gene nanoparticles. The activity was assessed using average increase in survival time, body weight, and solid tumor volume. All the specimens were then prepared as formalin-fixed and paraffin-embedded tissue sections for hematoxylin-eosin staining. The data showed that the nanoparticles could efficiently deliver plasmids into HeLa cells. The cytotoxicity of the HeLa cells was significantly increased by TRAIL/endostatin-loaded nanoparticles when compared with control groups. The use of TPGS in combination with TRAIL and endostatin had synergistic antitumor effects. In conclusion, the TRAIL/endostatin-loaded nanoparticles offer considerable potential as an ideal candidate for *in vivo* cancer gene delivery.

## Background

Cervical cancer caused by high-risk human papillomavirus (HPV) infection constitutes a major problem in women's health worldwide and is the fifth leading cause of cancer deaths among females [1,2]. Current treatments including surgery, chemotherapy, and radiotherapy remain to have several disadvantages, thereby often leading to recurrence [2]. Two prophylactic HPV vaccines (Gardasil and Cervarix) [3] can prevent most high-risk HPV infections and minimize the consequences of HPV-associated diseases. However, these vaccines are effective only in adolescents with no history of previous HPV infection and have not shown any therapeutic effects against current HPV infections or associated lesions [3]. Thus, there is an urgent need to develop new specific drugs and methods to treat cervical cancer. Tumor necrosis factor-related apoptosis-inducing ligand (TRAIL) is a type 2 transmembrane protein that causes apoptosis of target cells through the extrinsic apoptosis pathway. TRAIL belongs to a member of the tumor necrosis factor superfamily including tumor necrosis factor and Fas ligand [4]. The binding of tumor necrosis factor and Fas ligand leads to the damage of normal tissues in addition to their proapoptotic effect on transformed cells [5,6], thus limiting their clinical applications. Conversely, TRAIL is able to selectively induce apoptosis in transformed cells but not in most normal cells [4,7,8], making it a promising candidate for tumor therapy. Furthermore, tumor growth and progression rely upon angiogenesis [9-11]. Consequently, antiangiogenesis has also emerged as an attractive new strategy in the treatment of cancer [12-16]. Among these agents, endostatin, a 20-kDa C-terminal proteolytic fragment of collagen XVIII, has received the greatest attention [17]. It was found not only to be a potent inhibitor of angiogenesis *in vitro*, but also to have significant antitumor effects in a variety of xenograft-based cancer models and clinical trials [17]. These promising results lead to the rapid advance of this agent into the clinical test [17,18]. For instance, endostatin enhanced the anticancer effect of CCRT in a mouse xenograft model of cervical cancer [19]. Furthermore, the use of endostatin in combination with other anticancer agents such as gemcitabine had synergistic antitumor activities [20].

Delivery of therapeutic agents by gene therapy has been extensively studied in a broad range of diseases [21-24]. However, a recurrent problem in these therapies is the efficient delivery of the therapeutic DNA, RNA, or siRNA to the target cells. The key technological impediment to successful gene therapy is vector optimization. Thus, several strategies are being investigated to circumvent this problem such as adeno- or adeno-associated viruses [25]. However, clinical trials have demonstrated substantial obstacles to their use, such as immunogenicity and inflammatory potential [26]. Various non-viral delivery systems, including liposomes [27], dendrimers [28], polycationic polymers [29,30], and polymeric nanoparticles (NPs) [31] are under development to reduce or avoid immunogenicity and associated risks of toxicity [32]. In recent years, the development of non-viral vectors which could also increase transfection efficiency has received great attention [33-38]. Among these vector systems, nanoparticles offer a number of advantages that make them ideal candidates as vectors for specific gene therapy. Furthermore, nanoparticles for gene therapy can be simply prepared by conjugating DNA onto the nanoparticle surface. These nanoparticles could conveniently enter into the cell via endocytosis [39-41]. Bioconjugate techniques formed by the coating of cationic polymers onto the surface of nanoparticles have been employed for increasing the target gene complexing ability by regulation of cationic polymers coated onto the nanoparticles to optimize gene delivery [42-45]. To improve the transfection of plasmid DNA (pDNA) into cells, negatively charged pDNA and positively charged macromolecules can be linked by charge interaction. Polyethyleneimine (PEI), a representative cationic polymer, can be polyplexed to pDNA, and these polyplexes have been successfully used for gene transfection both *in vitro* and *in vivo*[46]. Although PEI is considered as one of the most efficient non-viral gene transfer agents, it has some limitations due to its cytotoxicity [47]. The hydrophilic polyethylene glycol (PEG) modification of PEI which was thought to create a more non-ionic surface of polyplexes was previously shown to reduce cytotoxicity [48].

In this research, a novel biodegradable diblock copolymer, TPGS-*b*-(PCL-*ran*-PGA), was successfully synthesized for nanoparticle formulation. We hypothesized that TPGS-*b*-(PCL-*ran*-PGA) nanoparticles modified with a polyplexed PEI could deliver TRAIL and/or endostatin to the target cells to treat xenograft models bearing HeLa cells. In the past decade, polycaprolactone (PCL) and its copolymers were used in a number of drug delivery devices. Due to the fact that PCL degrades at a slower rate than polyglycolide (PGA), poly-d,l-lactide, and its copolymers, it was therefore originally used in drug delivery devices that remain active for over 1 year and in slowly degrading suture materials [49]. Copolymerization of ε-caprolactone (ε-CL) with other monomers or fast degrading polymers, i.e., malic acid and PGA, could facilitate polymer degradation and control drug release. PGA is also not a perfect biomaterial for use in drug delivery systems [41]. The reason is that PGA has very high crystallinity (45% to 55%), has high melting temperature (about 220°C), and is insoluble in general solvent. Diblock copolymers and/or random copolymers offer the opportunity to combine properties of different parent homopolymers in a new material [2,41]. d-α-Tocopheryl polyethylene glycol 1000 succinate (TPGS), a water-soluble form of natural vitamin E, is synthesized by esterification of vitamin E succinate with PEG 1000. It was reported that TPGS was able to increase drug permeability through cell membranes by downregulating expression of P-glycoprotein (P-gp) and thus enhance absorption of drugs and reverse P-gp-mediated multidrug resistance in cancer cells [50,51]. Furthermore, TPGS effectively inhibited the growth of human lung carcinoma cells both *in vitro* and *in vivo*[52]. The superior antitumor activity of TPGS was associated with its increasing ability to induce apoptosis [52-54]. Synergistic anticancer effects could be obtained by the use of combinations of TPGS in the presence of other anticancer agents [53].

## Methods

### Materials

TPGS, glycolide (1,4-dioxane-2,5-dione), and stannous octoate were obtained from Sigma-Aldrich (St. Louis, MO, USA). Poly(vinyl alcohol) (PVA; 80% hydrolyzed), branched polyethyleneimine (MW ~ 25,000), and 3-(4,5-dimethylthiazol-2-yl)-2,5-diphenyl-2*H*-tetrazolium bromide (MTT) were also purchased from Sigma-Aldrich. ε-CL was from Acros Organics (Geel, Belgium). 4^′^,6-Diamidino-2-phenylindole dihydrocloride (DAPI) was obtained from VECTOR (Burlingame, CA, USA). Plasmid vectors pShuttle2, pIRES2-EGFP, and pDsRED-E1 were acquired from Invitrogen-Gibco (Carlsbad, CA, USA). Dulbecco's modified Eagles' medium (DMEM), fetal bovine serum (FBS), penicillin-streptomycin, and Dulbecco's phosphate-buffered saline (DPBS) were also from Invitrogen. All other chemicals and solvents used were of the highest quality commercially available.

### Synthesis and characterization of TPGS-*b*-(PCL-*ran*-PGA) diblock copolymer

TPGS-*b*-(PCL-*ran*-PGA) diblock copolymers were synthesized via ring opening copolymerization (ROP) of ε-CL, glycolide, and TPGS as described previously [41]. Briefly, weighted amounts of ε-CL, glycolide, and TPGS and one drop of stannous octoate were added into a dried glass ampoule. The ampoule was connected to a vacuum line, evacuated, sealed off, and placed in oil bath at 160°C. A slow and progressive viscosity increase of the bulk homogeneous mixture was always observed during the polymerization, and the copolymers were produced in over 3 h. After cooling to room temperature, the ampoule was opened, and the resulting copolymers were dissolved in dichloromethane (DCM) and then precipitated in excess cold methanol to remove unreacted TPGS and monomers. The final product was collected by filtration and dried at 45°C under vacuum.

Fourier transform infrared spectroscopy (FT-IR) (Nicolet Instrument Co., Madison, WI, USA) was employed to investigate the chemical structure of TPGS-*b*-(PCL-*ran*-PGA) copolymer. Briefly, the samples for FT-IR analysis were prepared by grinding 99% potassium bromide (KBr) with 1% copolymer and then pressing the mixture into a transparent disk in an evacuable die at sufficiently high pressure. The structure, number-averaged molecular weight (Mn) of the copolymer, and copolymer composition were determined by proton nuclear magnetic resonance (^1^H NMR) in CDCl_3_ at 300 Hz (Bruker ACF300, Madison, WI, USA). The weight-averaged molecular weight and molecular weight distribution were determined by gel permeation chromatography (Waters, Milford, PA, USA).

### Fabrication of TPGS-*b*-(PCL-*ran*-PGA) nanoparticles

The nanoparticles were fabricated by a multiple (W/O/W) emulsion solvent evaporation method [55]. In brief, 50 mg of TPGS-*b*-(PCL-*ran*-PGA) was emulsified with 2 ml DCM by sonication for 30 s. After the addition of an aqueous 6 ml of PVA solution (7% *w*/*v*), the emulsion was sonicated again for 25 s. The resulting double emulsion (W/O) was then poured into 100 ml of a 1% (*w*/*v*) PVA solution containing 2% isopropanol and then maintained under mechanical stirring for 1 h at 600 rpm. The residual DCM was then dried under vacuum. Then, aliquots of the nanoparticle suspension (1.8 ml) were washed twice with 20 mM 4-(2-hydroxyethyl)-1-piperazineethanesulfonic acid (HEPES)/NaOH (pH 7.0) by centrifugation (8,000 rpm, 10 min, 4°C) and then resuspended.

### Preparation of expression vectors

Human TRAIL and endostatin were amplified by polymerase chain reaction (PCR) using primers: hendostatin-F: 5^′^-GCTCTAGAgccaccatgggaattcatgcacagccaccgcgacttcc-3^′^ (*Xba*І), hendostatin-R: 5^′^-GGGGTACCttacttggaggcagtcatg-3^′^ (*Kpn*І); hTRAIL-F: 5^′^-GCTCTAGAgccaccatggtgagagaaagaggtcctcag-3^′^ (*Xba*І), hTRAIL-R: 5^′^-GGGGTACCttagccaactaaaaaggccc-3^′^ (*Kpn*І). The enzyme was digested and purified. PCR products were cloned into pShuttle2 vector (Clontech, Mountain View, CA, USA). The recombinant plasmids pShuttle2-TRAIL and pShuttle2-endostatin were verified by enzyme digestion and DNA sequencing. Protein expression was analyzed by Western blot as described below.

### Nanoparticle modification

The nanoparticles were formulated with an N/P ratio (ratio of the polymer nitrogen to the DNA phosphate) equal to TPGS-*b*-(PCL-*ran*-PGA) nanoparticle solution (0.2 ml) which was mixed with 2 mg of PEI in sterile HEPES-buffered saline. The PEI-modified TPGS-*b*-(PCL-*ran*-PGA) nanoparticle solution was then added to the plasmid DNA solution at different N/P ratios and vortexed gently. The pDNA-loaded TPGS-*b*-(PCL-*ran*-PGA)/PEI nanoparticles were incubated for 20 min at room temperature in sterile PBS.

### Nanoparticle characterization

The mean particle size and size distribution were measured by dynamic light scattering (Zetasizer Nano ZS90, Malvern Instruments, Malvern, UK). One milligram of nanoparticles was suspended in 3 ml of deionized (DI) water before measurement. Zeta potential of the nanoparticles was determined by laser Doppler anemometry (Zetasizer Nano ZS90, Malvern Instruments, Malvern, UK). Samples were prepared in PBS and diluted 1:3 with DI water to ensure that the measurements were performed under conditions of low ionic strength where the zeta potential of the nanoparticles can be measured accurately. The final concentration of the polymer was 1 mg/ml. The data were obtained with the average of three measurements. The particle morphology and size were examined by field emission scanning electron microscopy (FESEM).

### Loading efficiency of pDNA-loaded nanoparticles

The loading efficiency of pDNA-loaded nanoparticles was evaluated by measuring the amount of free (unloaded) pDNA in the nanoparticle solution. The TPGS-*b*-(PCL-*ran*-PGA)/PEI nanoparticles were centrifuged, and the supernatants were collected. DNA concentrations in the supernatants were measured using a UV spectrophotometer (Beckman, Fullerton, CA, USA) at 260 nm. Loading efficiency of pDNA in the nanoparticles was determined by subtracting the amount of pDNA recovered in the supernatants from the initial amount of pDNA added.

### *In vitro* release assay

To investigate the *in vitro* pDNA release, 5 mg of TPGS-*b*-(PCL-*ran*-PGA)/PEI nanoparticles (group HNP) was added in 1 ml of DPBS buffer (pH 7.4) and 25 mM sodium acetate buffer (pH 5.0), respectively, in an Eppendorf tube and kept in a shaker at 37°C. Samples were periodically withdrawn from each tube and centrifuged at 15,000 rpm for 15 min to obtain pellet nanoparticles. The supernatants were removed by aspiration and replaced with fresh buffer solution, and the nanoparticles were resuspended by vortexing and repeated pipetting to break up aggregated particles. The supernatants were kept at −40°C until analysis by UV spectroscopy.

### Gel retardation assay

Agarose gel electrophoresis was performed to determine the binding of pDNA with TPGS-*b*-(PCL-*ran*-PGA)/PEI nanoparticles. A series of different weight ratios (*w*/*w*) of pDNA to TPGS-*b*-(PCL-*ran*-PGA)/PEI nanoparticles was loaded on the agarose gel (10 ml of the sample containing 0.1 mg of pDNA). A 1:6 dilution of loading dye was added to each well, and electrophoresis was performed at a constant voltage of 100 V for 20 min in TBE buffer (4.45 mM Tris-base, 1 mM sodium EDTA, 4.45 mM boric acid, pH 8.3) containing 0.5 g/ml ethidium bromide. The pDNA bands were then visualized using a UV transilluminator at 365 nm.

### Cell culture

HeLa cells (ATCC, Manassas, VA, USA) were cultured in DMEM (pH 7.4) supplemented to contain 25 mM NaHCO_3_, 10 μg/ml streptomycin sulfate, 100 μg/ml penicillin G, and 10% (*v*/*v*) FBS. Cells were maintained at 37°C in an incubator with 5% CO_2_ and 95% air.

### Western blot

The cells were seeded into six-well tissue culture plates and allowed to attach to the substrate overnight. The cells were cultured at 37°C in an atmosphere of 5% CO_2_ in air and then rinsed twice and preincubated for 1 h with 2 ml of serum-free medium at 37°C. The recombinant plasmids pShuttle2-TRAIL and pShuttle2-endostatin were added at a particle concentration of 0.01 to 0.2 mg/ml and incubated for 1 to 4 h at 37°C. The cells were then washed three times with 1 ml ice-cold PBS (pH 7.4) to remove any free pShuttle2-TRAIL or pShuttle2-endostatin. The cells were continuously cultured in fresh complete medium for 48 h. The cells were lysed in cell lysis buffer containing PMSF for 30 min at 4°C. The lysate was then centrifuged at 13,000 rpm for 20 min at 4°C. The proteins were then separated by SDS-PAGE and transferred onto PVDF membranes. The membranes were blocked in a Tris-buffered saline with 0.1% Tween 20 (TBS-T) solution with 5% (*w*/*v*) non-fat dry milk and incubated overnight with primary antibodies at 4°C. The immunoreactive signals were detected with horseradish peroxidase-conjugated secondary antibodies followed by SuperSignal West Pico Chemiluminescent Substrate (Thermo Fisher Scientific, Rockford, IL, USA). For protein loading control, membranes were reprobed with anti-β-actin antibodies. For the *in vivo* studies, tumors were harvested, and the cell lysates were prepared and transferred to a clean microcentrifuge tube and centrifuged at 14,000 rpm for 30 min. The supernatant was subjected to Western blotting as described above.

### Cellular uptake of fluorescent TPGS-*b*-(PCL-*ran*-PGA)/PEI nanoparticles

The uptake of pIRES2-EGFP and/or pDsRED nanoparticles by HeLa cells were firstly observed by fluorescence microscopy. In brief, cells were preincubated in serum-free medium at 37°C for 1 h and then for 2 h in the presence of pIRES2-EGFP or pDsRED gene-loaded TPGS-*b*-(PCL-*ran*-PGA)/PEI nanoparticles (final particle concentration, 0.2 mg/ml). The samples were mounted in fluorescent mounting medium, and the fluorescence was observed under a fluorescence microscope (Leica DMI6000 B, Wetzlar, Germany).

For confocal laser scanning microscopy (CLSM) analysis, cells were preincubated in serum-free medium at 37°C for 1 h and then for 2 h in the presence of pIRES2-EGFP-loaded TPGS-*b*-(PCL-*ran*-PGA)/PEI nanoparticles (final particle concentration, 0.2 mg/ml). The cells were rinsed three times with cold PBS and then fixed by ethanol for 20 min. The nuclei were stained with DAPI for 30 min and washed twice with PBS. Finally, the cells were observed using a confocal laser scanning microscope (Fluoview FV-1000, Olympus Optical Co., Ltd., Tokyo, Japan).

### Cell viability

The cytotoxicity of gene nanoparticles was evaluated by the MTT assay. Briefly, HeLa cells were seeded at a density of 5 × 10^3^ cells/well in 100-μl culture medium into a 96-well plate and incubated overnight. The cells were incubated with various gene nanoparticles at 40 μg/ml nanoparticle concentration for 24 and 48 h, respectively. At designated time intervals, the medium was removed and 20 μl/well of 5 mg/ml MTT solution was added to each well. After 4 h of incubation at 37°C under a humidified atmosphere supplemented with 5% CO_2_ in air, MTT was taken up by active cells and reduced in the mitochondria to form insoluble purple formazan granules. Subsequently, the medium was discarded and the precipitated formazan was dissolved in dimethyl sulfoxide (150 ml/well), and optical density of the resulting solution was evaluated using a microplate spectrophotometer at a wavelength of 570 nm. The analytical assays were performed every day, and at least four wells were randomly taken for examination each time to determine viability based on the physical and biochemical properties of cells.

### *In vivo* studies

Female severe combined immunodeficient (SCID) mice of 15 to 20 g were provided by the Medical Experimental Animal Center of Guangdong Province (Guangzhou, China). Animal experiments were conducted under the protocol approved by the Institutional Animal Care and Use Committee of Tsinghua University. A subcutaneous xenograft nude mouse model was established. Six-week-old female nude mice (body weight = 18 ± 2 g) were inoculated subcutaneously with 1.5 to 2 × 10^6^ HeLa cells. When the average size of tumors reached approximately 100 mm^3^, the mice were randomly divided into six groups consisting of six mice each: PBS control, blank TPGS-*b*-(PCL-*ran*-PGA) nanoparticles (group DNP), blank TPGS-*b*-(PCL-*ran*-PGA)/PEI nanoparticles (group ENP), TRAIL-loaded TPGS-*b*-(PCL-*ran*-PGA)/PEI nanoparticles (group FNP), endostatin-loaded TPGS-*b*-(PCL-*ran*-PGA)/PEI nanoparticles (group GNP), and TRAIL- and endostatin-loaded TPGS-*b*-(PCL-*ran*-PGA)/PEI nanoparticles (group HNP). Each mouse in the treatment groups received a single dose of nanoparticles equivalent to 0.2 mg TPGS-*b*-(PCL-*ran*-PGA), 10 μg PEI, and 50 μg DNA (for TRAIL- or endostatin-loaded TPGS-*b*-(PCL-*ran*-PGA)/PEI nanoparticles, the amount of pDNA was equivalent to the amount of pShuttle2-TRAIL or endostatin plus pShuttle2). The groups were treated once every week with intratumoral injections of either PBS or gene nanoparticles. Tumor size was measured using a caliper, and the weight of each mouse was measured with a scale every 3 days until the end of the experiment. Tumor volume was calculated using the following formula: volume = length × width^2^/2. The mean tumor volume was used to construct a tumor growth curve to evaluate the therapeutic efficacy of gene nanoparticles. Tumor specimens were then prepared as formalin-fixed, paraffin-embedded sections for hematoxylin-eosin (H&E) staining.

### Statistical analyses

All experiments were repeated at least three times unless otherwise stated. *T* test statistical analysis was performed with SPSS 16.0 software (Chicago, IL, USA), with *P* < 0.05 considered to indicate a significant difference.

## Results and discussion

### Characterization of TPGS-*b*-(PCL-*ran*-PGA) diblock copolymer

**Figure 1:**
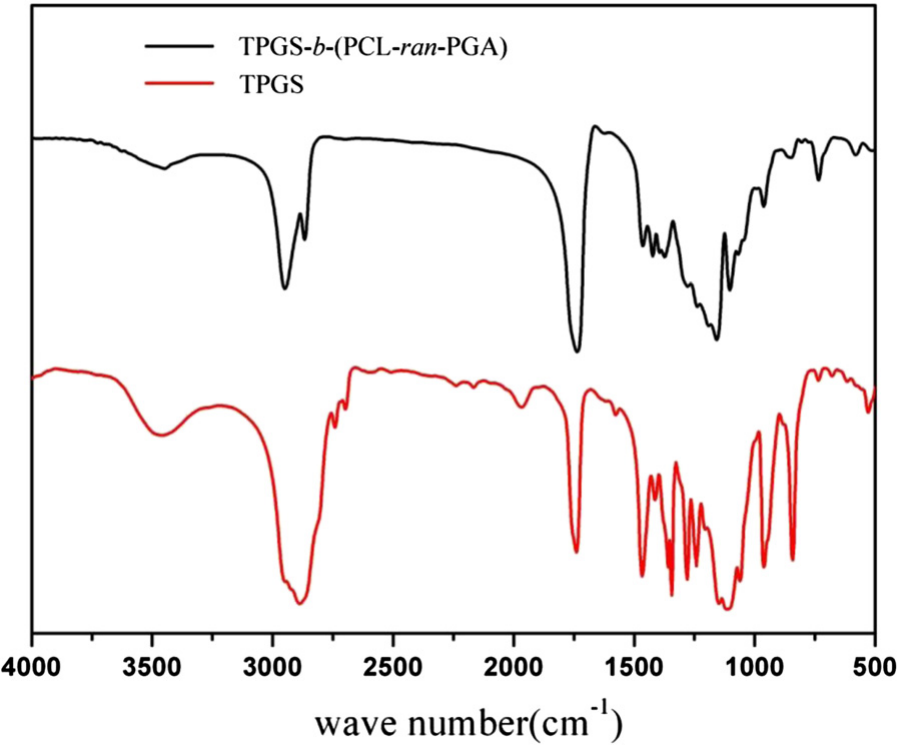
**FT-IR spectra of TPGS and TPGS-*b*-(PCL-*ran*-PGA) copolymer.**

**Figure 2:**
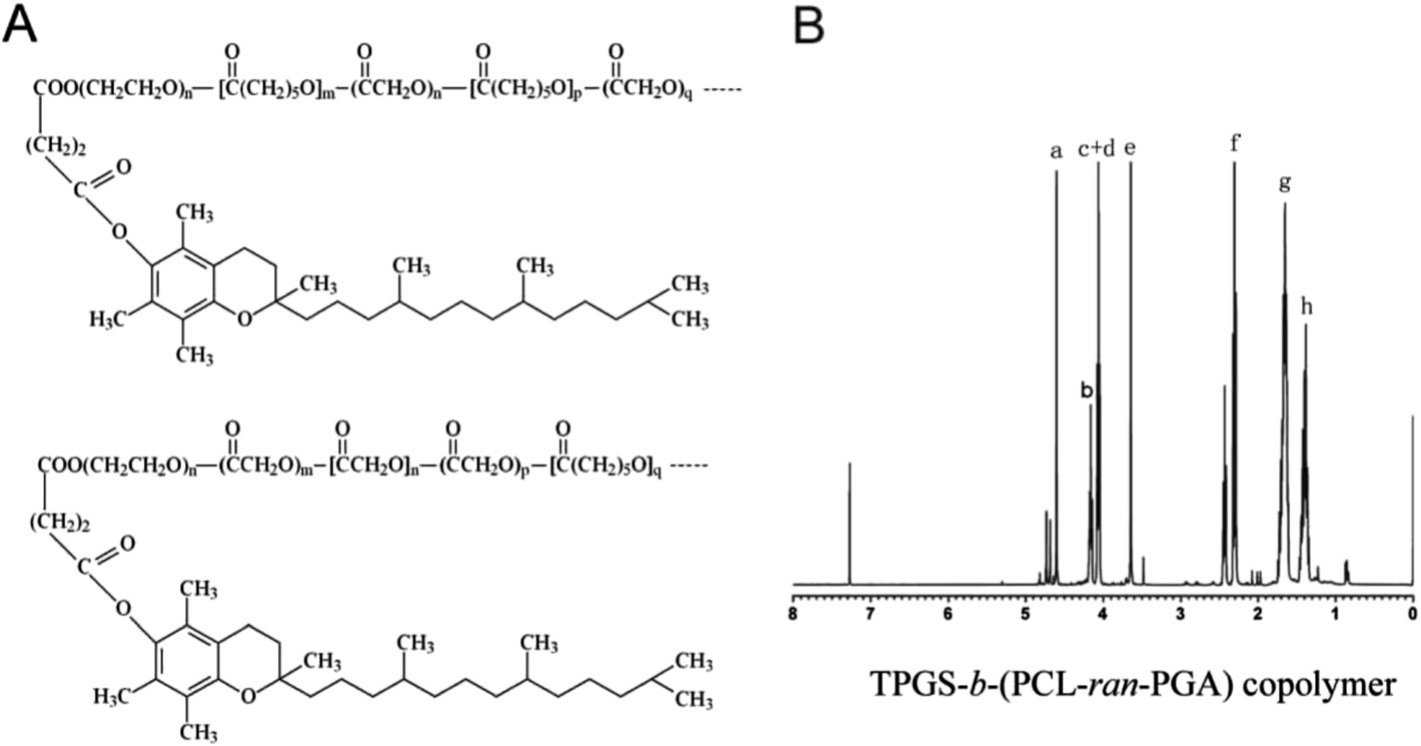
**Chemical structure (A) and typical **^**1**^**H NMR spectra (B) of TPGS-*b*-(PCL-*ran*-PGA) copolymer.**

The TPGS-*b*-(PCL-*ran*-PGA) diblock copolymer was successfully synthesized via ROP. FT-IR spectra of the TPGS-*b*-(PCL-*ran*-PGA) copolymer and TPGS are shown in Figure 1. The carbonyl band of TPGS was observed at 1,739 cm^−1^. For the TPGS-*b*-(PCL-*ran*-PGA) copolymer, the carbonyl band was shifted to 1,736 cm^−1^, which was also different with the carbonyl bands of PGA at 1,747 cm^−1^ and of PCL at 1,725 to 1,726 cm^−1^[56,57]. All the C-H stretching bonds are centered at 2,949 and 2,867 cm^−1^[56]. The absorption bands from 3,400 to 3,650 cm^−1^ are due to the terminal OH group, and that at 1,045 to 1,295 cm^−1^ is attributed to the C-O stretching [58]. Of those, the absorption bands from 1,105 to 1,242 cm^−1^ are attributed to the characteristic C-O-C stretching vibrations of the repeated -OCH_2_CH_2_ units of TPGS and the -COO bond stretching vibrations of GA and CL, respectively [56]. The band at 1,295 cm^−1^ has been used to investigate the crystallinity change in PCL [2]. All these signals indicate the TPGS-*b*-(PCL-*ran*-PGA) copolymer may be formed. The chemical structure of TPGS-*b*-(PCL-*ran*-PGA) copolymer is shown in Figure 2A. In order to further confirm the formation of the random copolymer, the ^1^H NMR spectrum is recorded and is shown in Figure 2B. The peak at 3.65 ppm (Figure 2, peak e) could be attributed to the -CH_2_ protons of the PEO part of TPGS [2,41]. The lower signals in the aliphatic zone belong to various moieties of vitamin E tails [2,41]. Peaks at 1.39 (h), 1.67 (g), 2.31 to 2.44 (f), and 4.06 ppm (d) are assigned to methylene protons in PCL units, respectively [2,41]. The difference between the two peaks at 4.06 (c) and 4.16 ppm (b) which indicated that two kinds of copolymers would be obtained was reasonable (shown in Figure 2). Furthermore, it was from the appearance of the two different peaks that we could conclude that both GA and CL monomers had copolymerized with TPGS monomers. The characteristic signal at 4.62 to 4.82 ppm (a) exists, which is attributed to the methylene (CH_2_) protons of the PGA units [41]. The molecular weight of the TPGS-*b*-(PCL-*ran*-PGA) copolymer was calculated by the use of the ratio between the peak areas at 4.06, 4.62 to 4.82, and 3.65 ppm. The Mn of the TPGS-*b*-(PCL-*ran*-PGA) copolymer was estimated to be 23,852. The Mn calculated from the gel permeation chromatograph was 25,811. It seemed that the molecular weight measured from NMR and GPC can confirm each other.

### Construction and expression of pShuttle2-TRAIL and pShuttle2-endostatin

**Figure 3:**
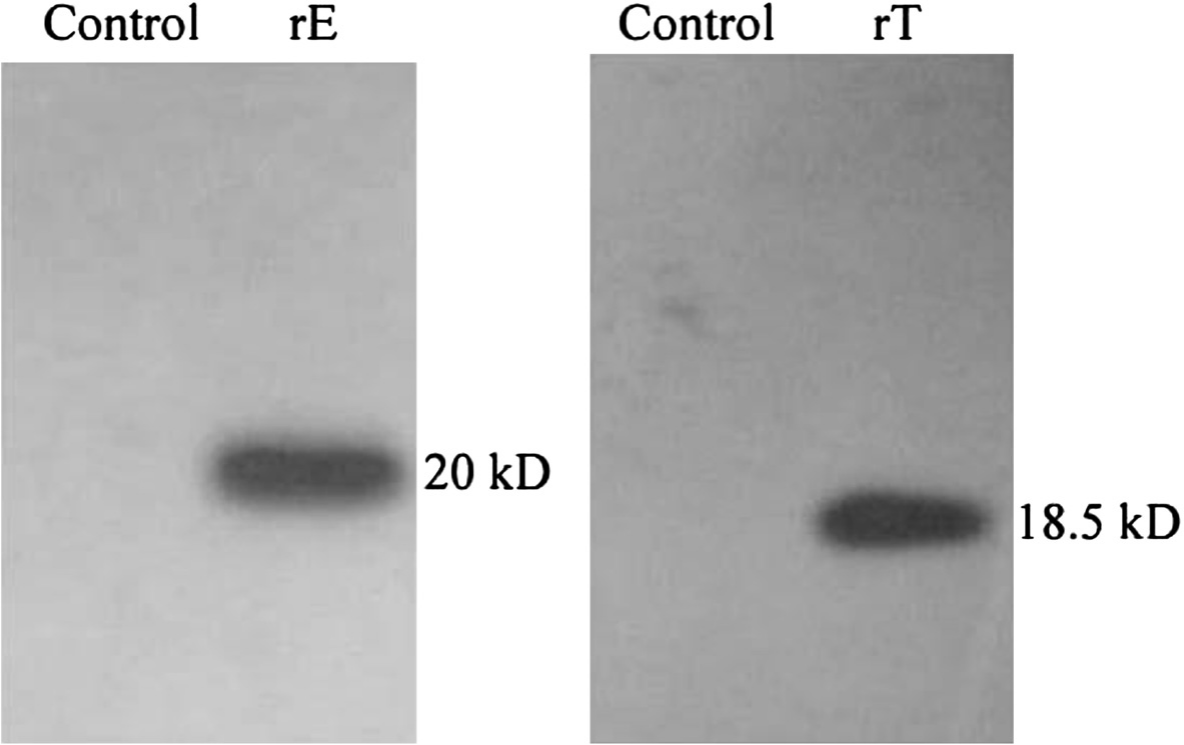
**Western blot analysis of recombined pShuttle2-endostatin and pShuttle2-TRAIL expression in 293 T cells.** Control: 293 T cells transfected by pShuttle2. rE: 293 T cells transfected by pShuttle2-endostatin. rT: 293 T cells transfected by pShuttle2-TRAIL.

Recombinant plasmids pShuttle2-TRAIL and pShuttle2-endostatin were verified by enzyme digestion and DNA sequencing. Protein expression of TRAIL and endostatin was analyzed by Western blot using cell lysate after transfection of HeLa cells using PEI (Figure 3). These results showed that pShuttle2-TRAIL and pShuttle2-endostatin were successfully constructed and expressed in HeLa cells.

### Characterization of nanoparticles

**Figure 4:**
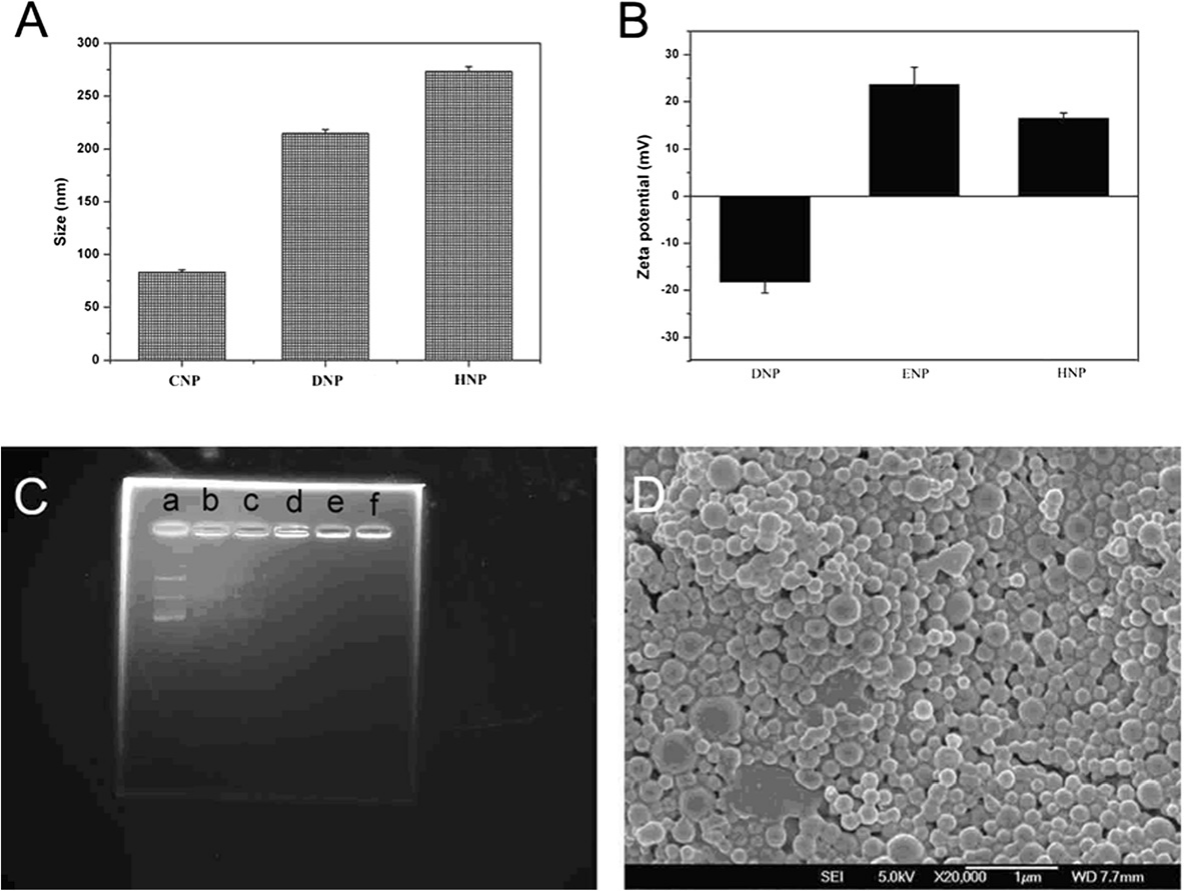
**Effects of PEI modification, binding of pDNA with TPGS-*b*-(PCL-*ran*-PGA)/PEI nanoparticles, and FESEM image of HNP.** (**A**) The effects of PEI modification on particle size. (**B**) The effects of PEI modification on surface charge. (**C**) The binding of pDNA with TPGS-*b*-(PCL-*ran*-PGA)/PEI nanoparticles determined by agarose gel electrophoresis. A series of different weight ratios (*w*/*w*) of pDNA to TPGS-*b*-(PCL-*ran*-PGA)/PEI nanoparticles was loaded on the agarose gel (a, pDNA/NPs = 1:0; b, pDNA/NPs = 1:4; c, pDNA/NPs = 1:10; d, pDNA/NPs = 1:20; e, pDNA/NPs = 1:20; f, pDNA/NPs = 1:20). (**D**) FESEM image of TRAIL- and endostatin-loaded TPGS-*b*-(PCL-*ran*-PGA)/PEI nanoparticles (HNP).

The effect of PEI modification on particle size was determined by dynamic light scattering (DLS; Table 1). The average hydrodynamic diameter of the polyplexed PEI/pDNA nanoparticles (CNP) was 83 nm, whereas the diameters of the unmodified TPGS-*b*-(PCL-*ran*-PGA) nanoparticles (DNP) and PEI-modified TPGS-*b*-(PCL-*ran*-PGA) nanoparticles (HNP) were approximately 215 and approximately 273 nm, respectively (Figure 4A). In addition, the surface charge (zeta potential) of the nanoparticles was determined by laser Doppler anemometry (Zetasizer Nano ZS90, Malvern Instruments, Malvern, UK; Table 1 and Figure 4B). Surface modification of the TPGS-*b*-(PCL-*ran*-PGA) nanoparticles significantly changed the zeta potential, suggesting that the negative surface charge of the TPGS-*b*-(PCL-*ran*-PGA) nanoparticles (DNP) was changed to a positive charge with PEI modification. Consistent with these results, a reduction in the positive charge for control PEI/TPGS-*b*-(PCL-*ran*-PGA) nanoparticles (ENP) was obtained because the TPGS-*b*-(PCL-*ran*-PGA) nanoparticles (DNP) was induced by the addition of negatively charged pDNA. The ability of all TPGS-*b*-(PCL-*ran*-PGA)/PEI nanoparticles to immobilize pDNA was confirmed by agrose gel electrophoresis (Figure 4C). In a recent report, the pDNA complexed to the polymeric (poly(lactic-*co*-glycolic acid (PLGA)) nanoparticles is in a condensed form, which could protect it against denaturation and allow to be efficiently taken up by MSCs. In addition, PLGA/PEI nanoparticles possessed the ability to condense DNA for protection against degradation [55]. Table 1 also shows the loading efficiencies of all PEI-modified gene nanoparticles (groups FNP, GNP, and HNP) which were above 60%.

**Table 1:** Characterization of nanoparticles

**Group**	**Size (nm)**	**Polydispersion**	**Zeta potential (mV)**	**Loading efficiency (%)**	**Gene**	**Polymer**
	**(*n *= 3)**		**(*n *= 3)**	**(*n *= 3)**		
ANP	72.11 ± 3.44	0.164	22.54 ± 3.47	83.4 ± 2.3	TRAIL	PEI
BNP	71.82 ± 5.18	0.156	21.58 ± 4.16	82.6 ± 1.9	Endostatin	PEI
CNP	83.02 ± 2.35	0.178	24.65 ± 2.78	78.3 ± 3.8	TRAIL/endostatin	PEI
DNP	215.06 ± 3.52	0.186	−18.25 ± 2.36	0	None	TPGS-*b*-(PCL-*ran*-PGA)
ENP	236.31 ± 1.44	0.201	23.65 ± 3.65	0	None	PEI/TPGS-*b*-(PCL-*ran*-PGA)
FNP	265.48 ± 4.40	0.229	19.45 ± 1.99	67.4 ± 4.3	TRAIL	PEI/TPGS-*b*-(PCL-*ran*-PGA)
GNP	245.48 ± 6.42	0.215	18.45 ± 2.67	64.6 ± 3.1	Endostatin	PEI/TPGS-*b*-(PCL-*ran*-PGA)
HNP	272.97 ± 4.68	0.245	16.54 ± 1.06	62.5 ± 0.9	TRAIL/endostatin	PEI/TPGS-*b*-(PCL-*ran*-PGA)

Surface morphology of the PEI-modified TPGS-*b*-(PCL-*ran*-PGA) nanoparticles was observed by FESEM. Figure 4D shows a typical FESEM image of the TPGS-*b*-(PCL-*ran*-PGA)/PEI nanoparticles. The morphologies of PEI-modified TPGS-*b*-(PCL-*ran*-PGA) particles were sphere-like nanoparticles in shape. The FESEM image further confirmed the particle size detected from DLS.

### *In vitro* release

**Figure 5:**
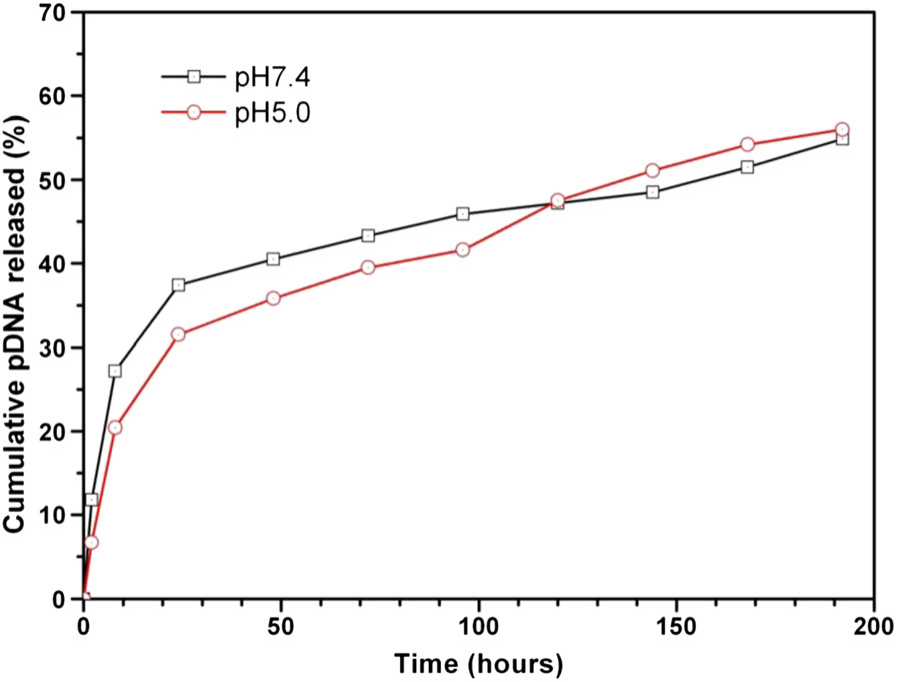
***In vitro *release profile of TRAIL- and endostatin-loaded TPGS-*b*-(PCL-*ran*-PGA)/PEI nanoparticles at pH 7.4 and 5.0.**

The timing of nanoparticle degradation and DNA release appears to have a significant modulating impact on the gene expression [59]. We compared the *in vitro* release of pDNA from PEI-modified TPGS-*b*-(PCL-*ran*-PGA) nanoparticles at pH 5.0 and pH 7.4. pDNA release was determined by measuring UV absorption at 260 nm at specific time points. The data showed that 40.5% of the loaded pDNA was released rapidly from PEI-modified TPGS-*b*-(PCL-*ran*-PGA) nanoparticles within 48 h at pH 7.4, followed by sustained release until day 8 (Figure 5). This fact may be due to the dependency of the TPGS-*b*-(PCL*-ran*-PGA) degradation on the external conditions. It was reported that at low pH values, cleavage of the ester linkage of the polyester backbone such as PLGA was catalyzed to accelerate the polymer degradation. However, at pH 7.4, the release kinetics of pDNA was similar with that at pH 5.0. PEI, which is a hydrophilic molecule located at the surface of the TPGS-*b*-(PCL*-ran*-PGA) matrix, may hasten degradation of the nanoparticles by increasing hydration and thereby promoting hydrolysis [30].

### Cellular uptake of TPGS-*b*-(PCL-*ran*-PGA)/PEI nanoparticles

**Figure 6:**
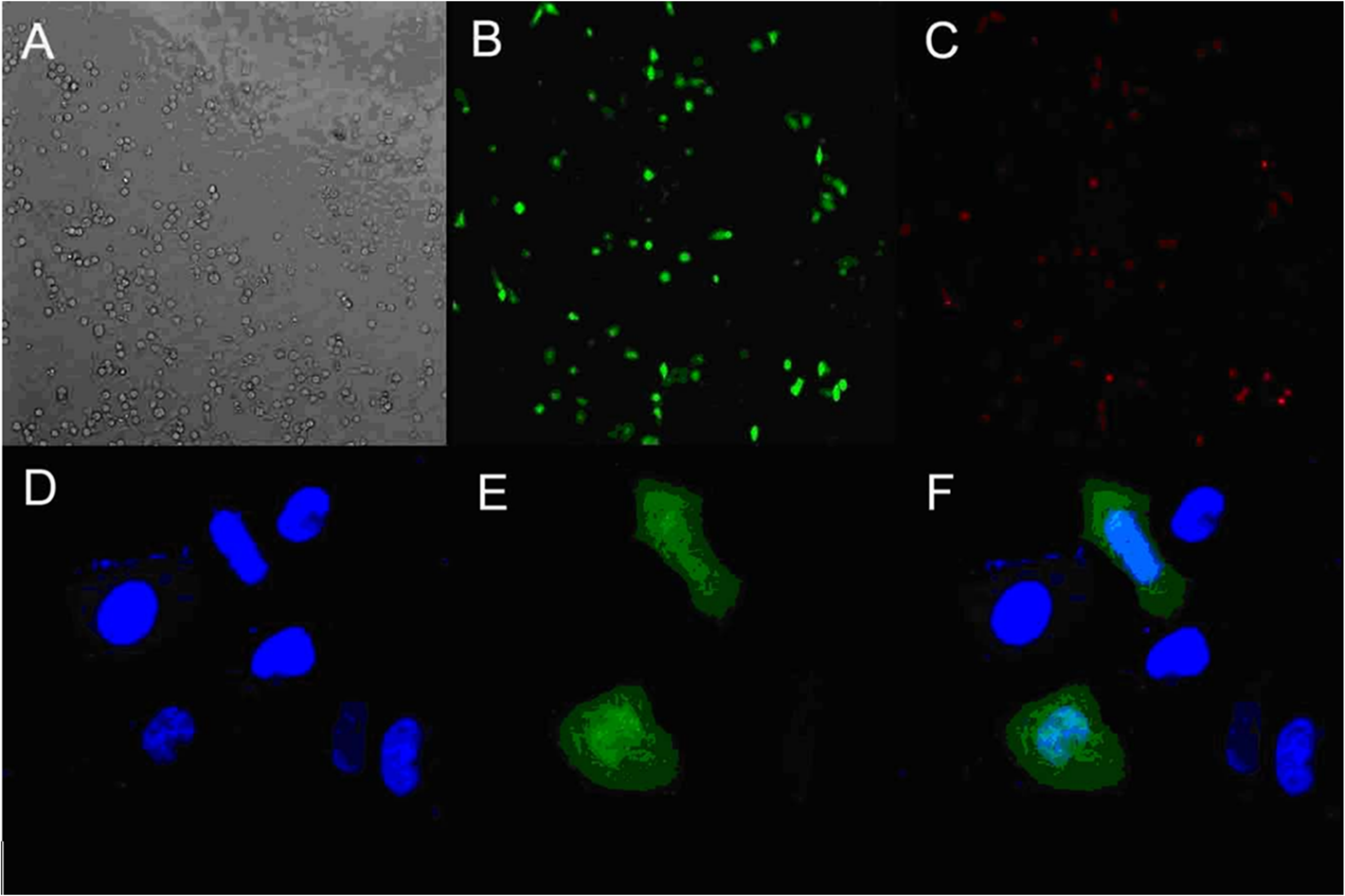
**Fluorescence and confocal laser scanning microscopy images of HeLa cells after incubation.** (**A** to **C**) The fluorescence microscopy images of HeLa cells after incubation with pIRES2-EGFP-loaded and pDsRED-loaded TPGS-*b*-(PCL-*ran*-PGA)/PEI nanoparticles. (**D** to **F**) Confocal laser scanning microscopy images of HeLa cells after incubation with pIRES2-EGFP-loaded TPGS-*b*-(PCL-*ran*-PGA)/PEI nanoparticles at 37.0°C. The cells were stained by DAPI (blue), and the pIRES2-EGFP-loaded TPGS-*b*-(PCL-*ran*-PGA)/PEI nanoparticles are in green. The cellular uptake was visualized by overlaying images obtained using DAPI filter and FITC filter: (**D**) from DAPI channel, (**E**) from FITC channel, (**F**) from combined DAPI channel and FITC channel.

To determine cellular uptake of nanoparticles, HeLa cells were incubated with TPGS-*b*-(PCL-*ran*-PGA)/PEI nanoparticles. Figure 6 shows the fluorescence imaging of HeLa cells after incubation with pIRES2-EGFP-loaded and pDsRED-loaded TPGS-*b*-(PCL-*ran*-PGA)/PEI nanoparticles. As can be seen in Figure 6, HeLa cells showed strong green (Figure 6B) and red (Figure 6C) fluorescence, indicating that pIRES2-EGFP-loaded and pDsRED-loaded TPGS-*b*-(PCL-*ran*-PGA)/PEI nanoparticles could be efficiently internalized into the cells.

CLSM images showed that the fluorescence of the pIRES2-EGFP-loaded TPGS-*b*-(PCL-*ran*-PGA)/PEI nanoparticles (green) was located around the entire cell including the nucleus area (blue, stained by DAPI) (Figure 6D,E,F), which further confirmed that the nanoparticles could efficiently deliver plasmids into HeLa cells.

### Cell viability of gene nanoparticles

**Figure 7:**
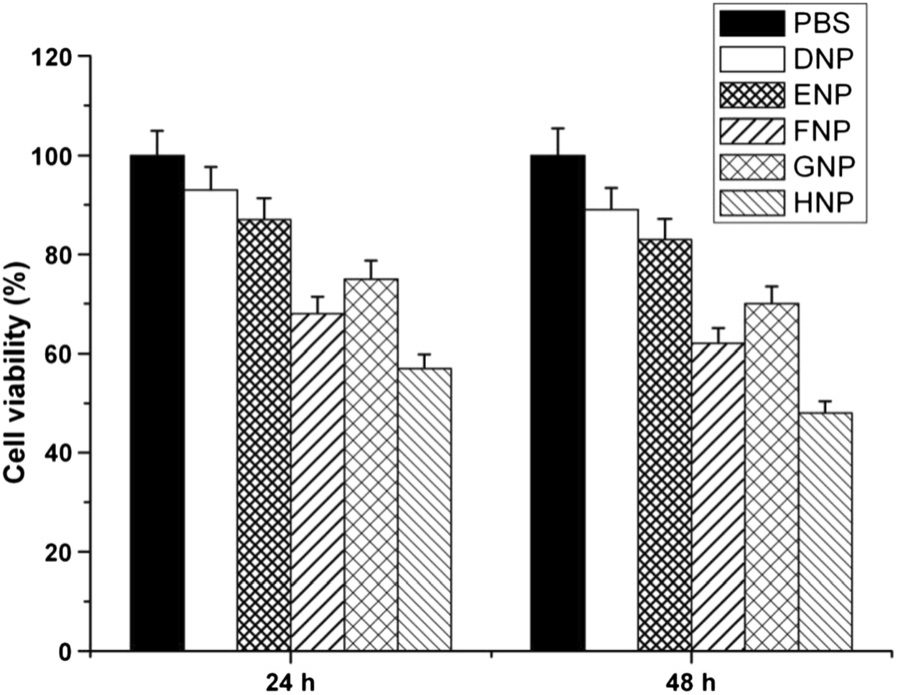
**Viability of HeLa cells cultured with various nanoparticles in comparison with that of PBS.** After 24- and 48-h incubation. (*n* = 5).

Cytotoxicity of all gene nanoparticles (groups FNP, GNP, and HNP), blank TPGS-*b*-(PCL-*ran*-PGA) nanoparticles (group DNP), and blank TPGS-*b*-(PCL-*ran*-PGA)/PEI nanoparticles (group ENP) was compared to that of PBS by the MTT assay. Figure 7 shows the *in vitro* viability of HeLa cells cultured with various nanoparticles (groups DNP, ENP, FNP, GNP, and HNP) and PBS (*n* = 5) after 24- and 48-h incubation. The results showed that all gene-loaded TPGS-*b*-(PCL-*ran*-PGA)/PEI nanoparticles appeared to have significant cytotoxicity than other control nanoparticles (*P* < 0.05). Especially, TRAIL- and endostatin-loaded TPGS-*b*-(PCL-*ran*-PGA)/PEI nanoparticles (group HNP) had much more cytotoxicity (*P* < 0.01). The higher cytotoxicity of TRAIL- and endostatin-loaded TPGS-*b*-(PCL-*ran*-PGA)/PEI nanoparticles (group HNP) may be attributed to synergistic antitumor effects of TRAIL and endostatin and the degradation and release of TPGS from TPGS-*b*-(PCL-*ran*-PGA). It was reported that the superior antitumor activity of TPGS was due to its increasing ability to induce apoptosis [52-54]. Synergistic antitumor activities could be obtained by the use of combinations of TPGS and other anticancer drugs [53].

### *In vivo* studies

**Figure 8:**
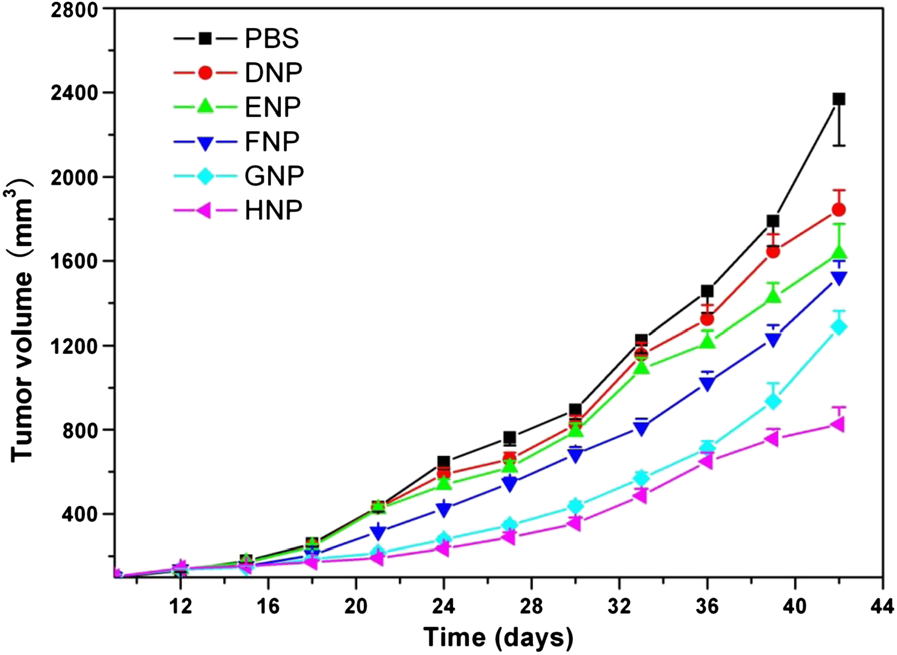
Antitumor effect of various nanoparticles in comparison with that of PBS.

**Figure 9:**
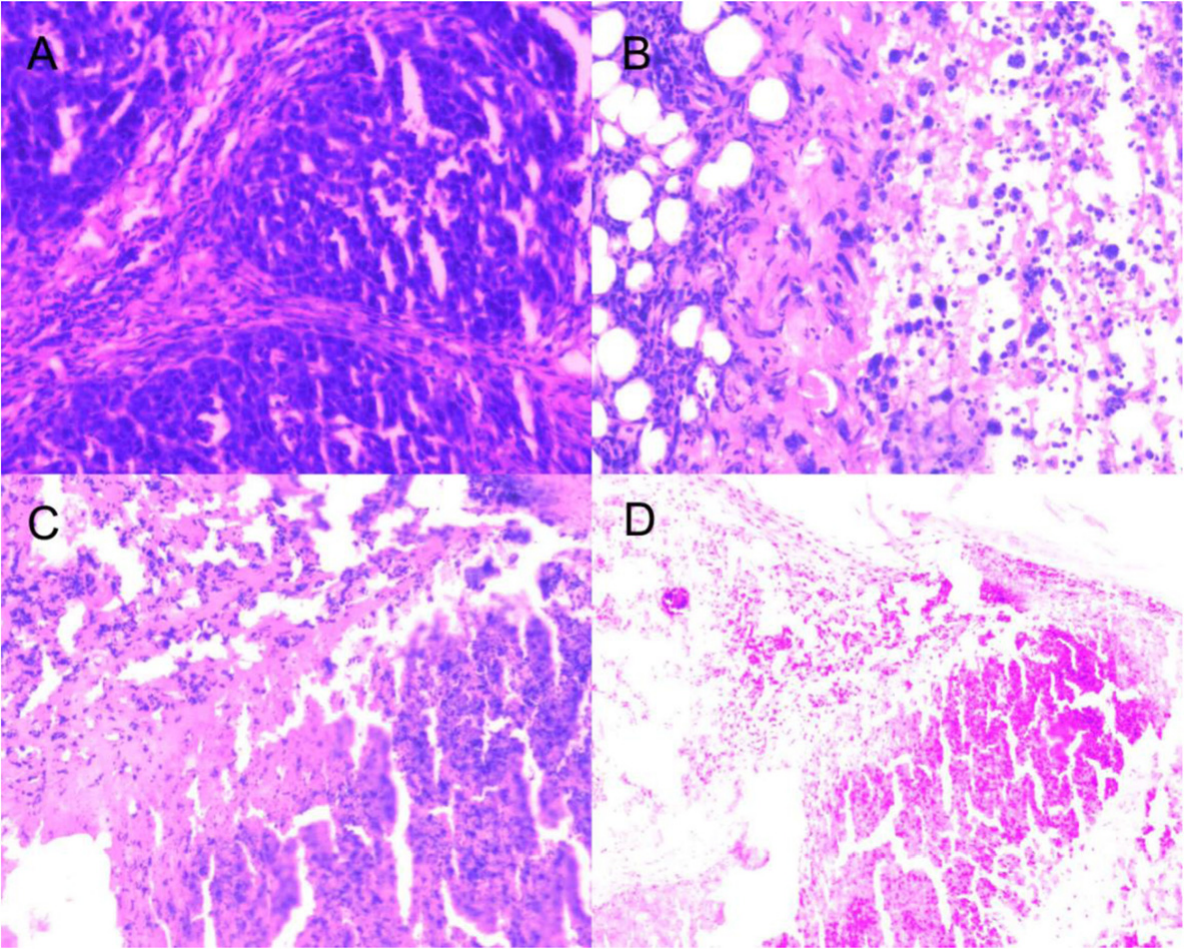
**Representative H&E staining of tumors.** Treated with PBS (**A**), TRAIL-loaded TPGS-*b*-(PCL-*ran*-PGA)/PEI nanoparticles (**B**), endostatin-loaded TPGS-*b*-(PCL-*ran*-PGA)/PEI nanoparticles (**C**), and TRAIL and endostatin-loaded TPGS-*b*-(PCL-*ran*-PGA)/PEI nanoparticles (**D**).

The antitumor efficacy of all gene nanoparticles (groups FNP, GNP, and HNP) was further evaluated on SCID mice of an average body weight of approximately 17.8 g and an average initial tumor volume of approximately 103 mm^3^. The data showed that the mean survival time of mice treated with TRAIL/endostatin-loaded nanoparticles was significantly longer than that of the control mice, whereas body weight among these groups had no statistical difference (*P* > 0.05). The average tumor growth volume was shown in Figure 7 in comparison with those of the PBS control, blank TPGS-*b*-(PCL-*ran*-PGA) nanoparticles (group DNP), and blank TPGS-*b*-(PCL-*ran*-PGA)/PEI nanoparticles (group ENP). It can be seen from Figure 8 that TRAIL- and endostatin-loaded TPGS-*b*-(PCL-*ran*-PGA)/PEI nanoparticles (group HNP) significantly slowed down the tumor growth of mice in comparison with the PBS control and other nanoparticles. Compared with the PBS control, blank TPGS-*b*-(PCL-*ran*-PGA) nanoparticles (group DNP) could also have slight anticancer efficacy. This phenomenon may be due to the degradation and release of TPGS from the TPGS-*b*-(PCL-*ran*-PGA) copolymer. It was reported that TPGS could also have superior anticancer efficacy by inducing apoptosis [52-54]. By considering the overall slope of all the curves in Figure 8, it can be concluded that the TRAIL- and endostatin-loaded TPGS-*b*-(PCL-*ran*-PGA)/PEI nanoparticles (group HNP) have significant advantages than controls and single gene-loaded nanoparticles (groups FNP and GNP) in suppressing tumors. Thus, we could conclude that synergistic antitumor activities could be obtained by the use of combinations of TRAIL, endostatin, and TPGS. As shown in Figure 9, the images of H&E staining also indicated that tumor growth treated by TRAIL- and endostatin-loaded TPGS-*b*-(PCL-*ran*-PGA)/PEI nanoparticles (group HNP) was significantly inhibited in comparison with that of the PBS control. In conclusion, the TRAIL/endostatin-loaded nanoparticles offer considerable potential as an ideal candidate for *in vivo* cancer gene delivery.

In future studies, we will investigate the combined effect of TRAIL/endostatin gene therapy and chemotherapeutic agents such as doxorubicin, docetaxel, and floxuridine, encapsulated in TPGS-*b*-(PCL-*ran*-PGA) nanoparticles, in different cervical cancer cell lines and animal models in order to make clear whether a combination of TRAIL/endostatin gene therapy and chemotherapy will have enhanced antitumor activity. We hypothesize that surface modification of TPGS-*b*-(PCL-*ran*-PGA) nanoparticles with polyethyleneimine may also be a promising and useful drug and gene co-delivery system.

## Conclusions

For the first time, a novel TPGS-*b*-(PCL-*ran*-PGA) nanoparticle modified with polyethyleneimine was applied to be a vector of TRAIL and endostatin for cervical cancer gene therapy. The data showed that the nanoparticles could efficiently deliver plasmids into HeLa cells and the expression of TRAIL and endostatin was verified by RT-PCR and Western blot analysis. The cytotoxicity of the HeLa cells was significantly increased by TRAIL/endostatin-loaded nanoparticles when compared with control groups. Synergistic antitumor activities could be obtained by the use of combinations of TRAIL, endostatin, and TPGS. The images of H&E staining also indicated that tumor growth treated by TRAIL- and endostatin-loaded TPGS-*b*-(PCL-*ran*-PGA)/PEI nanoparticles was significantly inhibited in comparison with that of the PBS control. In conclusion, the TRAIL/endostatin-loaded nanoparticles offer considerable potential as an ideal candidate for *in vivo* cancer gene delivery.

## Competing interests

The authors declare that they have no competing interests.

## Authors' contributions

YZ carried out the *in vivo* studies and drafted the manuscript. HC carried out the cell studies. XZ carried out the preparation of nanoparticles. YoZ carried out the characterization of nanoparticles. XX carried out the *in vitro* drug release studies. ZL participated in the *in vivo* studies. DG participated in the design of the study and performed the statistical analysis. LM conceived of the study and participated in its design and coordination. All authors read and approved the final manuscript.
